# Silicon-Doped Carbon Dots Crosslinked Carboxymethyl Cellulose Gel: Detection and Adsorption of Fe^3+^

**DOI:** 10.3390/gels10050285

**Published:** 2024-04-23

**Authors:** Zhengdong Zhao, Yichang Jing, Yuan Shen, Yang Liu, Jiaqi Wang, Mingjian Ma, Jiangbo Pan, Di Wang, Chengyu Wang, Jian Li

**Affiliations:** 1 Key Laboratory of Bio-Based Material Science and Technology, Ministry of Education, Northeast Forestry University, Harbin 150040, China; zhaozd6866@163.com (Z.Z.); yichangjing0302@163.com (Y.J.); jlaushawn@yeah.net (Y.S.); hljqaliuyang@163.com (Y.L.); w1531565456@163.com (J.W.); 18847042762@163.com (M.M.); pjb0725@163.com (J.P.); wangcy@nefu.edu.cn (C.W.); nefujianli@163.com (J.L.); 2 College of Material Science and Engineering, Northeast Forestry University, Harbin 150040, China

**Keywords:** silicon-doped carbon dots crosslinked gel, carboxymethyl cellulose, trivalent iron detection, trivalent iron adsorption

## Abstract

The excessive emission of iron will pollute the environment and harm human health, so the fluorescence detection and adsorption of Fe^3+^ are of great significance. In the field of water treatment, cellulose-based gels have attracted wide attention due to their excellent properties and environmental friendliness. If carbon dots are used as a crosslinking agent to form a gel with cellulose, it can not only improve mechanical properties but also show good biocompatibility, reactivity, and fluorescence properties. In this study, silicon-doped carbon dots/carboxymethyl cellulose gel (DCG) was successfully prepared by chemically crosslinking biomass-derived silicon-doped carbon dots with carboxymethyl cellulose. The abundant crosslinking points endow the gel with excellent mechanical properties, with a compressive strength reaching 294 kPa. In the experiment on adsorbing Fe^3+^, the theoretical adsorption capacity reached 125.30 mg/g. The introduction of silicon-doped carbon dots confers the gel with excellent fluorescence properties and a good selective response to Fe^3+^. It exhibits a good linear relationship within the concentration range of 0–100 mg/L, with a detection limit of 0.6595 mg/L. DCG appears to be a good application prospect in the adsorption and detection of Fe^3+^.

## 1. Introduction

In recent years, water pollution caused by heavy metals such as Fe^3+^ has become a serious concern [1]. Excessive accumulation of Fe^3+^ in the human body can pose significant health hazards, thus necessitating its removal and detection in water [2]. Currently, the methods for treating metal ion pollution include chemical precipitation, electrolysis, ion exchange, membrane separation, etc. However, these methods are not only expensive but can also cause secondary pollution [3]. Among the numerous reported methods for removing and detecting metal ion pollution, the adsorption method has the advantages of simplicity, high efficiency, low cost, recyclability of adsorbents, and environmental friendliness [4], while fluorescence detection has the advantages of low cost and easy operation [5]. Both of them have broad application prospects and are worthy of attention [6]. Cellulose-based materials have been widely studied for heavy metal adsorption due to their excellent physicochemical properties, mechanical performance, and renewability. Gel materials with cellulose and its derivatives as the framework have attracted significant attention due to their high surface area and porous structure [7,8]. Cellulose derivatives such as carboxymethyl cellulose, cellulose acetate, and ethyl cellulose contain oxygen-containing functional groups that can form gel membranes through physical crosslinking via van der Waals forces and hydrogen bonds [9,10]. However, gels relying solely on physical crosslinking exhibit poor mechanical properties, limiting their application range. Chemically crosslinked gels, on the other hand, possess stronger stability and mechanical properties, thus finding wider applications [11]. Nevertheless, commonly used crosslinking agents, such as glutaraldehyde and epichlorohydrin, are toxic and incompatible with environmentally friendly cellulose-based materials [7,12]. Therefore, there is a need to explore novel and green crosslinking agents. Additionally, the low photoluminescence properties of cellulose and its derivatives often do not meet the requirements for fluorescent sensing, necessitating the introduction of emission sources [13,14,15].

Carbon dots, a new class of luminescent materials, possess strong photoluminescence and stability, making them widely applicable in the field of fluorescent sensing. Furthermore, the rich active groups on the surface of carbon dots allow them to serve as clean crosslinking agents for gel formation with framework materials. Therefore, combining carbon dots with cellulose derivatives can not only form gels but also improve the optical properties of the cellulose derivatives [16,17,18]. Additionally, the excellent mechanical properties of cellulose derivatives can address the challenges associated with the difficulty in processing and shaping carbon dots due to their size, achieving a win–win situation [19]. Among various carbon dots precursors, carboxymethyl chitosan, a derivative of natural polysaccharides, is chosen for synthesizing carbon dots due to its widespread availability and renewability. However, carbon dots prepared from polysaccharides as carbon sources typically exhibit poor luminescent properties. To enhance the luminescent performance of carbon dots, amino silane doping can be employed to adjust the band structure of carbon dots, leading to the synthesis of silicon-doped carbon dots with strong luminescent properties. Simultaneously, the introduction of additional amino groups increases the reactive sites [20].

In this work, silicon-doped carbon dots (Si-CDs) were prepared through a one-step hydrothermal method and subsequently chemically crosslinked with carboxymethyl cellulose through amide bonds to form a gel material (DCG). The numerous hydrophilic active groups, including hydroxyl, amino, and carboxyl, present within DCG offer numerous chelate sites for Fe^3+^, conferring upon DCG the capability to chemically adsorb Fe^3+^ in aqueous solutions. Moreover, Si-CDs exhibit excellent fluorescent properties and specific responsiveness to Fe^3+^. Therefore, DCG demonstrates promising application potential in the adsorption and detection of Fe^3+^.

## 2. Results and Discussion

### 2.1. Si-CD Structural Analysis

**Figure 1:**
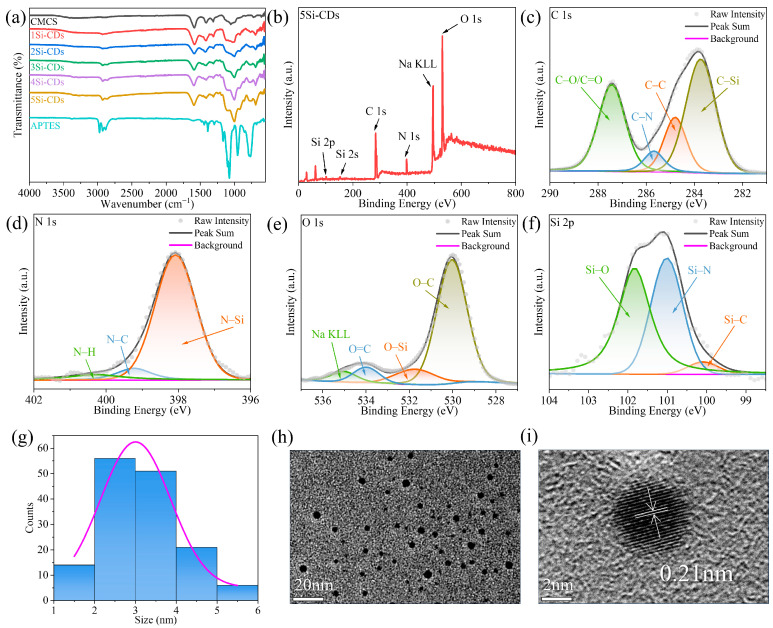
(**a**) FTIR spectra of CMCS, Si-CDs, and APTES. XPS spectra of 5Si-CDs: (**b**) full spectrum, (**c**) C 1s, (**d**) N 1s, (**e**) O 1s, and (**f**) Si 2p. (**g**) Size distribution histogram, (**h**) TEM image, and (**i**) HRTEM image of 5Si-CDs. Arrows and lines indicate one of the lattice spacings.

FTIR spectra were used to detect the occurrence of the reaction, and the FTIR spectra of CMCS, APTES, and Si-CDs are shown in Figure 1a. The pure carboxymethyl chitosan powder shows stretching vibrational absorption bands of hydroxyl and amino groups at 3410 cm^−1^. The peaks near 2900 and 2870 cm^−1^ are attributed to the stretching vibration of C-H. The peak at 1052 cm^−1^ is attributed to the bending vibration of C-H of the pyranose ring. The peaks at 1412 and 1587 cm^−1^ are attributed to the asymmetric stretching vibration peaks and symmetric stretching vibrational peaks, which are associated with the presence of -COONa and -COOH [21]. The peak at 1310 cm^−1^ is attributed to the asymmetric vibrational peak of C-N [22]. Comparing the infrared absorption curves of CMCS, new peaks appeared in the curves of Si-CDs, namely, the asymmetric stretching vibration and symmetric stretching vibration peaks of Si-O-Si at 1002 and 690 cm^−1^, the peak of Si-O-C at 1188 cm^−1^, and the bending vibration peak of Si-OH at 923 cm^−1^. The peaks at 1002 and 690 cm^−1^ are the asymmetric stretching vibration and symmetric stretching vibration peaks of Si-O-Si, which proved that Si-OH produced by APTES hydrolysis is dehydrated and condensed to form siloxane structure again [23]. The disappearance of the -CH_3_ peak at 2974 cm^−1^ present in APTES proves that the hydrolysis of APTES is complete [24]. The peaks related to -COO- are retained in Si-CDs, indicating that Si-CDs have -COOH and -COONa. Moreover, 1310 cm^−1^ asymmetric C-N vibrational peaks still exist, and more obvious bending vibrational peaks of -NH_2_ are produced at 3352 and 3284 cm^−1^, which proves that the amino group of APTES is retained on the surface of Si-CDs after the reaction [24].

In order to further investigate the molecular structure and functional group composition of Si-CDs, XPS tests were carried out with 5Si-CDs, and peak fitting of high-resolution XPS spectra was performed. The full XPS spectra in Figure 1b shows that the Si-CDs contain the elements C, N, O, Si, and Na. In the C 1s spectra in Figure 1c, C-Si, C-C, C-N, and C-O/C=O correspond to 283.74, 284.80, 285.68, and 287.43 eV [25,26]. In the N 1s spectra in Figure 1d, N-Si, N-C, and N-H correspond to 398.08, 399.23, and 400.30 eV [27,28,29]. In the O 1s spectra in Figure 1e, O-C, O-Si, and O=C correspond to 530.04, 531.73, and 533.99 eV [30]. In the Si 2p spectra in Figure 1f, Si-C, Si-N, and Si-O correspond to 100.09, 101.01, and 101.83 eV [29]. Comparing the XPS full spectra of CMCS in Figure S1 with the C 1s, N 1s, and O 1s spectra, the appearance of N-Si, O-Si, and Si elemental peaks proves the successful introduction of APTES. The results of the XPS spectra are in agreement with those of the FTIR spectra, which proves that the surface of Si-CDs possesses an abundance of reactive groups, such as hydroxyl, amino, and carboxyl groups.

The corresponding histogram of size distribution in Figure 1g and Figure S2 shows that the particle size is in line with the particle size range of carbon dots [31]. The TEM image of Si-CDs in Figure 1h and Figure S2 shows that they are spherical, without any aggregation, and well dispersed in water. The HRTEM image with higher resolution in Figure 1i and Figure S2 shows diffraction streaks of Si-CDs with a spacing of 0.21 nm, corresponding to the (100) crystallographic plane of graphitic carbon [32], which suggests that the core of Si-CDs is mainly carbon. Combined with the results of HRTEM, FTIR, and XPS, it can be hypothesized that the Si-CDs are a structure of the Si-O-Si skeleton encapsulating a graphitic carbon core [24,33].

### 2.2. DCG Structural Analysis

**Figure 2:**
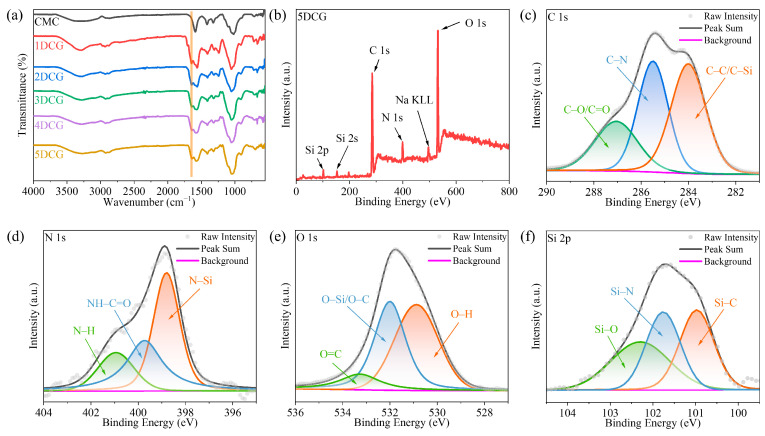
(**a**) FTIR spectra of CMC and DCG. XPS spectra of 5DCG: (**b**) full spectrum, (**c**) C 1s, (**d**) N 1s, (**e**) O 1s, and (**f**) Si 2p.

The FTIR spectra of CMC and DCG are shown in Figure 2a. In the infrared spectra of pure CMC powder, the telescopic vibrational peak of -OH is at 3285 cm^−1^ [34]. The peaks at 2917 and 2871 cm^−1^ are attributed to the asymmetric and symmetric telescopic vibrational absorption peaks of the C-H bond in -CH_2_- and -CH_3_ [35]. The peaks at 1590 and 1414 cm^−1^ are attributed to the antisymmetric -COO- and symmetric telescopic vibrational absorption peaks [36]. The telescopic vibrational absorption peak of the glycosidic bond on the cellulose pyran ring is at 1020 cm^−1^ [37]. In the infrared spectra of DCG, the C-H asymmetric stretching vibrational peaks and symmetric stretching vibrational peaks at 2922 and 2878 cm^−1^ are blue shifted. The intensity of the amino peaks near 3400 cm^−1^ increases, and a new stretching vibrational absorption peak of -CO-NH- appears at 1640 cm^−1^ [38]. The formation of chemical bonding connections between Si-CDs and CMCs is suggested, indicating that the amino group of Si-CDs has undergone a reaction with the carboxyl group of CMC to form an amide bond, thereby confirming the successful synthesis of DCG. Similarly, the appearance of the stretching vibration absorption peak of -CO-NH- at 1640 cm^−1^ also demonstrates the successful synthesis of SiDCG.

In order to further investigate the molecular structure and functional group composition of DCG, it was subjected to XPS tests. The full XPS spectra of DCG in Figure 2b shows that DCG contains elements such as C, N, O, and Si. In the C 1s spectra in Figure 2c, C-Si/C-C, C-N, and C-O/C=O correspond to 284.00, 285.50, and 287.05 eV [39], respectively, and the appearance of distinct C-Si and C-N peaks indicates the successful introduction of Si-CDs. In the N 1s spectra in Figure 2d, N-Si, NH-C=O, and N-H correspond to 398.80, 399.73, and 400.94 eV [29,40], respectively, and the appearance of NH-C=O peaks suggests that the Si-CDs and the CMC are chemically linked by amide bonds. In the O 1s spectra in Figure 2e, O-H, O-C/O-Si, and O=C correspond to 530.88, 532.00, and 533.27 eV, respectively. In the Si 2p spectra in Figure 2f, Si-C, Si-N, and Si-O correspond to 100.97, 101.76, and 102.30 eV, respectively [29]. Comparing the XPS full spectra, C1s spectra, and O1s spectra of CMC in Figure S3, the appearance of peaks related to N and Si proves the successful reaction of DCG with Si-CDs. The XPS results are in agreement with the FTIR results, which prove the successful synthesis of DCG.

**Figure 3:**
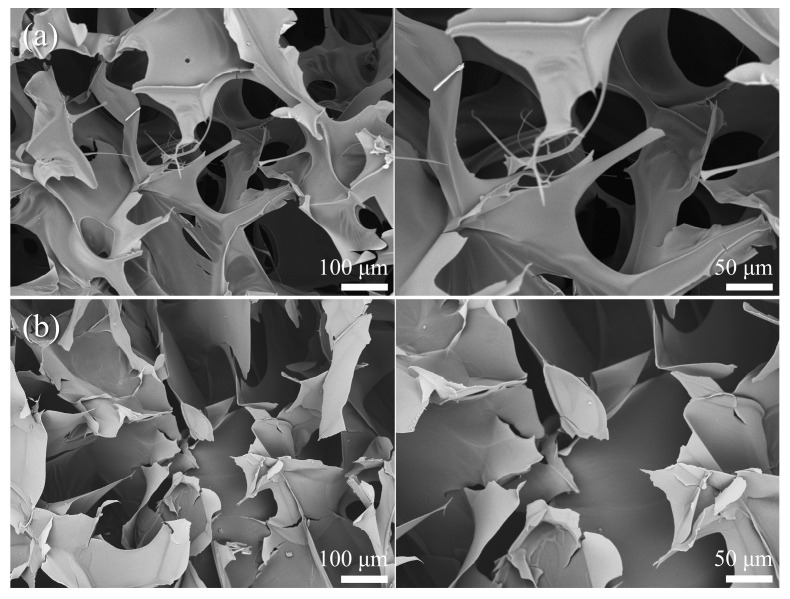
SEM image of (**a**) freeze-dried CMC and (**b**) 5DCG.

In order to investigate the microstructural changes of CMC after crosslinking with Si-CDs, SEM tests were performed on them, taking 5DCG as an example, and the results are shown in Figure 3. The carboxymethyl cellulose gel obtained by the freeze-drying method has a porous microstructure, and it remains porous after crosslinking with Si-CDs, which does not lead to the collapse of the gel structure or the closure of the micropores, and the lamellae become more flat [16]. In order to investigate the crystal structures of CMC and DCG, XRD was performed, and the results are shown in Figure S4, where the peak around 22° corresponds to the (110) crystal plane of graphitized carbon. Overall, both CMC and DCG are amorphous structures [41].

### 2.3. Mechanical Properties and Thermal Stability Analysis of DCG

**Figure 4:**
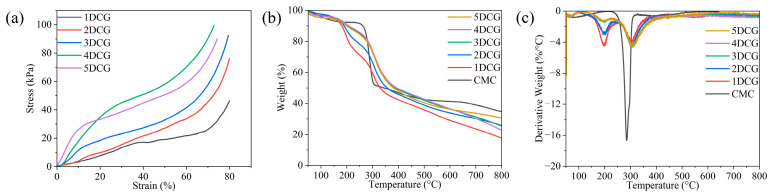
(**a**) Stress–strain curve of DCG. (**b**) TG and (**c**) DTG curves of CMC and DCG.

In order to investigate the mechanical properties of DCGs (1DCG, 2DCG, 3DCG, 4DCG, and 5DCG) obtained by crosslinking silicon-doped carbon dots with different molar ratios of APTES with carboxymethyl cellulose, the compressive stress-strain curves were determined by a universal mechanical testing machine, and the results are shown in Figure 4a. The calculated Young’s modulus was shown in Table 1. With the increase in the proportion of APTES in Si-CDs, the elastic deformation of the gels due to the external force gradually becomes smaller, and they are able to withstand greater stress without significant deformation. The results of Young’s modulus calculations are shown in the table. APTES enhances the content of amino groups on the surface of Si-CDs and increases the crosslinking point with carboxymethyl cellulose, and the Young’s modulus of DCG gradually increases from 35.3233 to 294 kPa. High Young’s modulus can better maintain the original structure in the process of application. Therefore, 5DCG has better stability in application due to its good mechanical properties [42].

In order to investigate the thermal stability and thermal decomposition of CMC and DCG, thermogravimetric analysis was carried out, and the results are shown in Figure 4b,c. The weight loss of CMC is divided into three stages: the first stage from 50 to 215 °C is caused by the evaporation of free and bound water adsorbed by CMC, and the weight loss is about 8%; the second stage from 315 to 330 °C is caused by the degradation and carbonization of the sugar chains of CMC; and the third stage from 330 to 800 °C is caused by the degradation and carbonization of the sugar chains of CMC and the sugar-containing chains of CMC, and the weight loss is about 42%. The weight loss in the second stage is caused by the degradation and carbonization of the sugar chains of CMC, and the weight loss in this stage is about 42%. The weight loss in the third stage from 330 to 800 °C is caused by the gradual and complete carbonization of the sugar chains and oxygen-containing groups of CMC, and the weight loss in this stage is about 7% [43]. The thermal decomposition behaviors of 1DCG, 2DCG, 3DCG, 4DCG, and 5DCG are similar, so the 5DCG is discussed in detail as an example. The weight loss of 5DCG is mainly divided into three stages. The weight loss in the first stage from 50 to 110 °C is caused by the successive evaporation of free and bound water physically adsorbed by the DCG. The weight loss in this stage is about 5%, which is lower than the percentage of weight loss of CMC. The reason for this change is the reduction of carboxyl groups after crosslinking CMC with Si-CDs, which reduces the percentage of bound water. The weight loss in the second stage from 110 to 230 °C is caused by the breakage of amide bonds at the crosslinking point, and the weight loss in this stage is about 8% [44]. The weight loss in the third stage from 230 to 800 °C is caused by the gradual and complete degradation and carbonization of the sugar chain and the oxygen-containing groups, and the weight loss in this stage is about 57% [39].

### 2.4. Analysis of the Fluorescence Properties of DCG

**Figure 5:**
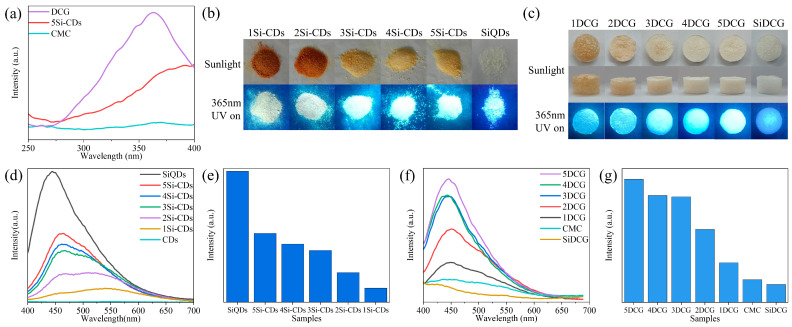
(**a**) The fluorescence excitation spectra of 5DCG, 5Si-CDs, and CMC (λ_em_ = 445 nm). (**b**) Photographs under daylight and 365 nm UV light, (**d**) fluorescence emission spectra under excitation light at 360 nm, and (**e**) histogram of fluorescence emission peak intensity of Si-CDs and SiQDs powders. (**c**) Photographs under daylight and 365 nm UV light, (**f**) histogram of fluorescence emission peak intensity, and (**g**) fluorescence emission spectra under excitation light at 360 nm of DCG and SiDCG.

The fluorescence excitation spectra of 5DCG, 5Si-CDs, and CMC are shown in Figure 5a. The optimal excitation wavelength of 5DCG is approximately 360 nm; therefore, a 360 nm light source was used for the subsequent excitation of fluorescence emission spectra. The photographs of Si-CDs and SiQDs under daylight and 365 nm UV light are shown in Figure 5b. Under daylight, 1Si-CDs and 2Si-CDs powders appear yellowish-brown, while 3Si-CDs, 4Si-CDs, and 5Si-CDs powders appear yellow, and the SiQD powder appears pure white. The higher the proportion of CMCS, the deeper the color of the powder. Under 365 nm UV light, all Si-CDs exhibit visible blue fluorescence emission, and the higher the proportion of APTES, the higher the brightness of the blue fluorescence of Si-CD powder. To investigate the fluorescence properties of solid powders, fluorescence spectra under 360 nm excitation light were tested, and the results are shown in Figure 5d. The fluorescence intensity statistics of the emission peaks are shown in Figure 5e. The wavelength of the emission peak is approximately 445 nm, and its intensity order is consistent with visual observation. The main and top view photographs of the DCG and SiDCG in daylight and under UV light at 365 nm are shown in Figure 5c. Under daylight, 1DCG, 2DCG, 3DCG, 4DCG, and 5DCG gradually change from yellow to white. Under 365 nm UV light, the DCG all have bright blue fluorescence emission with increasing brightness, while the brightness of SiDCG is relatively dim. In order to investigate the fluorescence properties of DCG and SiDCG, the fluorescence spectra under 360 nm excitation light were tested, and the results are shown in Figure 5f. The fluorescence emission peak of DCG is around 445 nm. The statistics of fluorescence intensity at the emission peak are shown in Figure 5g, and the fluorescence intensities of 1DCG to 5DCG increase. Comparing the fluorescence spectra of DCG and CMC, the introduction of Si-CDs greatly enhances the fluorescence performance of CMC. The introduction of Si-CDs not only acts as a crosslinking agent but also brings excellent fluorescence performance. As the mechanical and fluorescence properties of 5DCG are optimal, 5DCG was used in the following study, referred to as DCG for brevity.

### 2.5. DCG Detection of Fe^3+^ Concentration

**Figure 6:**
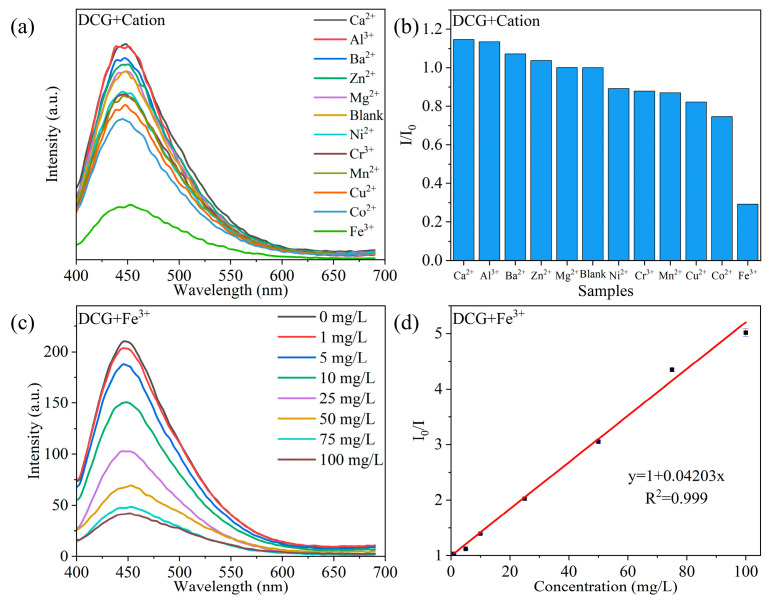
Ion screening and Fe^3+^ detection. (**a**) Effect of different metal cations on the fluorescence emission spectra of DCG (λ_ex_ = 360 nm). (**b**) Change of fluorescence emission peak intensity (I: fluorescence emission peak intensity after addition of metal cation, I_0_: initial fluorescence intensity of DCG). (**c**) Fluorescence emission spectra of DCG after addition of different concentrations of Fe^3+^ (λ_ex_ = 360 nm). (**d**) Relationship between I_0_/I and Fe^3+^ concentration and linear fitting.

To demonstrate the selectivity of DCG in detecting metal ions, the influence of several common metal cations on the fluorescence emission of DCG was explored. To eliminate the interference of anions in the experiment, chloride salts were used. As shown in Figure 6a, several ions (Ca^2+^, Al^3+^, Ba^2+^, Zn^2+^, Mg^2+^, Ni^2+^, Cr^3+^, Mn^2+^, Cu^2+^, Co^2+^, and Fe^3+^) do not shift the fluorescence emission peak of DCG but only affect its intensity. As shown in Figure 6b, Ca^2+^, Al^3+^, Ba^2+^, Zn^2+^, and Mg^2+^ cause aggregation of Si-CDs, slightly enhancing the fluorescence emission intensity of DCG, while Ni^2+^, Cr^3+^, Mn^2+^, Cu^2+^, Co^2+^, and Fe^3+^ quench the fluorescence emission of DCG. Among them, Fe^3+^ exhibits the most significant quenching effect [45,46], indicating that DCG could be used for specific detection of Fe^3+^. As shown in Figure 6c, changes in the concentration of Fe^3+^ do not shift the fluorescence emission peak of DCG. Therefore, the intensity of the fluorescence emission peak of DCG can be used to represent the concentration of Fe^3+^. As shown in Figure 6d, there is a good linear relationship between the concentration of Fe^3+^ and the change in fluorescence intensity of DCG in the range of 0–100 mg/L, with a linear correlation coefficient of R^2^ = 0.999. It highly corresponds to the single quenching mechanism of the Stern–Volmer equation (static quenching or dynamic quenching) [47]. The calculated detection limit is 0.6595 mg/L [48], which is much lower than China’s wastewater quality standards for discharge to municipal sewers (GB/T 31962–2015) of 10.0 mg/L [49], thus making it capable of meeting general testing needs.

### 2.6. Analysis of Fe^3+^ Adsorption Properties of DCG

**Figure 7:**
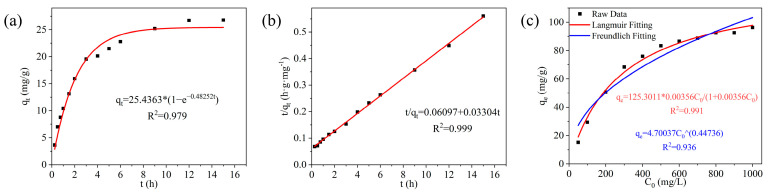
The performance of DCG in adsorbing Fe^3+^. (**a**) Pseudo-first-order kinetic fitting, (**b**) pseudo-second-order kinetic fitting, and (**c**) adsorption isotherm fitting.

Adsorption kinetics is an important physical quantity to study the adsorption rate. In order to study and analyze the adsorption kinetics of DCG and to determine the rate-controlling steps in the adsorption process, the pseudo-first-order kinetic model and the pseudo-second-order kinetic model were used in this study to simulate the adsorption of Fe^3+^ [44]. The pseudo-first-order kinetic and pseudo-second-order kinetic equations are shown in Equations (1) and (2), respectively. The fitting results are shown in Figure 7a,b, and the related parameters are shown in Table 2. The correlation coefficients (R^2^) of the pseudo-first-order kinetic equation and pseudo-second-order kinetic equation for the adsorption of ferric ions by the Gs1el are 0.979 and 0.999, respectively. The pseudo-first-order kinetic adsorption rate constant (k_1_) for the adsorption of Fe^3+^ by the DCG is 0.48252 h^−1^, and the pseudo-second-order kinetic adsorption rate constant (k_2_) is 0.01790 g·mg^−1^·h^−1^. The correlation coefficients show that the adsorption of Fe^3+^ by DCG is more in accordance with the pseudo-second-order kinetic equation, indicating that the adsorption of Fe^3+^ by DCG is mainly carried out by chemisorption, which is the main rate-controlling step in the adsorption process. In the 100 mg/L Fe^3+^ solution, the theoretical equilibrium adsorption capacity of DCG for Fe^3+^ according to the pseudo-second-order kinetic equation is 30.2633 mg/g.

(1) $$\ln\left(q_{e}-{\;q}_{t}\right){=\mathrm{lnq}}_{e}-{\;k}_{1}t,$$

(2) $$\frac{t}{q_{t}}=\frac{1}{k_{2}q_{e}^{2}}+\frac{t}{\mathrm{qe}},$$

To investigate the interaction between DCG and Fe^3+^, this study employed the Langmuir and Freundlich adsorption isotherm models to fit and analyze the experimental data for equilibrium adsorption isotherms. The Langmuir and Freundlich model equations are presented in Equations (3) and (4), respectively. The fitting results are shown in Figure 7c, and the related parameters are shown in Table 3. Compared to the fitting equation of the Freundlich model, the Langmuir model demonstrates a higher correlation coefficient (R^2^) (greater than 0.99), indicating that the adsorption of iron ions from the solution by the gel aligns with the Langmuir model, which can be considered monolayer adsorption [50]. The theoretical equilibrium adsorption capacity of DCG for Fe^3+^, according to the Langmuir model equation, is 125.3011 mg/g.

(3) $$\frac{C_{e}}{q_{e}}=\frac{C_{e}}{q_{e}}+\frac{1}{{q\times K}_{L}},$$

(4) $${\mathrm{lnq}}_{e}{=\mathrm{lnK}}_{F}+\frac{1}{n}{\mathrm{lnC}}_{e},$$

A comparison of the Fe^3+^ adsorption capacity and detection limits of the DCG with other cellulose-based materials is summarized in Table 4. DCG exhibits significant dual functionality, characterized by a high adsorption capacity of 125.3011 mg/L and a low detection limit for Fe^3+^ of 0.6595 mg/L. Its exceptional performance suggests a promising application in the adsorption and monitoring of Fe^3+^ in environmental pollution scenarios.

## 3. Conclusions

In summary, a gel DCG with a three-dimensional network structure was successfully synthesized by crosslinking Si-CDs with CMC. The crosslinking structure of the gel DCG brought about by Si-CDs exhibits excellent mechanical properties, with the compressive strength increasing as the APTES doping in Si-CDs increases, reaching a maximum of 294 kPa. Si-CDs confers excellent fluorescence properties on the gel, enabling specific responsiveness to Fe^3+^. DCG demonstrates a superior responsivity towards Fe^3+^ compared to other metal ions, thus possessing the potential for application in the detection of authentic Fe^3+^-containing wastewater samples with complex metal ion compositions. A good linear relationship was observed within the concentration range of 0–100 mg/L, with a detection limit of 0.6595 mg/L. It highly corresponds to the single quenching mechanism of the Stern–Volmer equation. Additionally, the adsorption of Fe^3+^ by DCG is a chemical and monolayer adsorption process, with a calculated theoretical maximum adsorption capacity of 125.30 mg/g. Therefore, DCG exhibits potential applications in the adsorption and detection of Fe^3+^.

## 4. Materials and Methods

### 4.1. Materials

Carboxymethyl chitosan (CMCS, MW: 100,000–200,000, DS ≥ 80%), carboxymethyl cellulose (CMC, MW = 250,000, DS = 1.2, μ = 400–800 mPa·s), (3-Aminopropyl)triethoxysilane (APTES, purity 98.0%), N-Hydroxysuccinimide (NHS, purity 98.0%), and zinc chloride (ZnCl_2_) were purchased from Shanghai Maclean Biochemical Technology Co. (Shanghai, China). N-(3-Dimethylaminopropyl)-N′-ethylcarbodiimide hydrochloride (EDC, purity 98.0%) was purchased from Aladdin Reagent Co. (Shanghai, China). Ferric chloride (FeCl_3_) was purchased from Tianjin Hengxing Chemical Preparation Co. (Tianjin, China). Calcium chloride (CaCl_2_) was purchased from Tianjin Reference Chemical Reagent Co. (Tianjin, China). Barium chloride (BaCl_2_) was purchased from the Tianjin Dongli district Tianda chemical reagent factory (Tianjin, China). Magnesium chloride (MgCl_2_) was purchased from Tianjin Ruijinte Chemical Co. (Tianjin, China). Manganese chloride (MnCl_2_) was purchased from Tianjin Xinbote Chemical Co. (Tianjin, China). Copper chloride (CuCl_2_), cobalt chloride (CoCl_2_), nickel chloride (NiCl_2_), aluminum chloride (AlCl_3_), and chromic chloride (CrCl_3_) were purchased from Tianjin Fuchen Chemical Reagent Co. (Tianjin, China). Anhydrous ethanol and 5-sulfosalicylic acid were purchased from Tianjin Tianli Chemical Reagent Co. (Tianjin, China). These chemicals were analytically pure and were not further purified prior to use. Deionized water produced by Clever-Q30 UT (Shanghai Kehuai Instruments Co., Shanghai, China) was used in this study.

### 4.2. Preparation of the Si-CDs and SiQDs

First, 0.5 g of CMCS powder was dissolved in 50 mL of deionized water, and APTES (1:1, 2:1, 3:1, 4:1, and 5:1 molar ratio to CMCS structural units) was added and stirred at room temperature for 10 min. Then, the reaction was transferred to a 100 mL PTFE-lined stainless-steel autoclave and placed in a blower oven at a constant temperature of 180 °C for 6 h [29]. After being cooled to room temperature, the reaction was filtered using a 0.22 μm micropore membrane and then dialyzed with a 1000 Da dialysis bag for 3 days. The dialysate was concentrated by a rotary evaporator and freeze-dried to obtain a yellow powder. 1Si-CDs, 2Si-CDs, 3Si-CDs, 4Si-CDs, and 5Si-CDs with blue fluorescence emission are collectively referred to here as Si-CDs. Next, 3 g of APTES was dissolved in 50 mL of deionized water, and SiQDs [24] were prepared by the same method for performance comparison.

### 4.3. Preparation of the DCG and SiDCG

First, 0.1 g of CMC powder was dissolved in 7 mL of deionized water, followed by the addition of 1 mL of a 50 mg/mL aqueous solution of 1Si-CDs, 2Si-CDs, 3Si-CDs, 4Si-CDs, and 5Si-CDs. This was followed by stirring for 10 min and then sonication for 10 min to ensure Si-CDs were uniformly dispersed between the CMC chains. Then, 1 mL of freshly prepared 0.5 mmol/L EDC aqueous solution was added under stirring, and 1 mL of freshly prepared 0.5 mmol/L NHS aqueous solution was added after stirring for another 5 min. After 24 h of reaction at room temperature, the unreacted material was removed by dialysis in a 50% ethanol aqueous solution, and freeze-drying yielded yellow to white 1DCG, 2DCG, 3DCG, 4DCG, and 5DCG with blue fluorescence emission. They are collectively referred to here as DCG. The gel obtained by crosslinking SiQDs with CMC is referred to as SiDCG.

### 4.4. Structure Characterizations

Fourier transform infrared spectroscopy (FTIR) images were obtained using the PerkinElmer Frontier spectrometer, produced by PerkinElmer, Waltham, MA, USA, with a measurement range of 4000–550 and a resolution of 2 cm^−1^. The UV–visible diffuse reflection spectra and UV–visible absorption spectra were measured using the UV–visible spectrophotometer TU-1950, produced by PERSEE, Beijing, China. X-ray photoelectron spectroscopy (XPS) images were obtained using K-alpha, an X-ray photoelectron spectrometer produced by Thermo Fisher Scientific, Waltham, MA, USA. Transmission electron microscope (TEM) images were obtained with the transmission electron microscope JEM-2100, produced by Japan Electronics Co., Ltd, Tokyo Metropolis, Japan. Fluorescence spectra were recorded with the LS-55, a fluorescence spectrophotometer produced by PerkinElmer, USA. Thermogravimetric analysis (TGA) was carried out using the STA 6000-SQ8 analyzer, produced by PerkinElmer in the USA, heated from 40 to 800 °C in a nitrogen atmosphere at a rate of 10 °C/min. X-ray diffraction (XRD) patterns were measured using the X-ray diffractometer X’Pert^3^ Powder, produced by PANalytical, Almelo, Netherlands. The surface morphology of the gel was observed using a TM3030 scanning electron microscope manufactured by Hitachi, Tokyo, Japan, with a test accelerating voltage of 5 kV. The mechanical properties were obtained by the universal mechanical testing machine INSTRON5942, produced by INSTRON, Boston, MA, USA.

### 4.5. Ion Screening and Fe^3+^ Detection Experiments

Ion screening: An aqueous solution of 1 mol/L metal salt was configured, and 0.5 mL was taken and added dropwise to the surface of DCG. Then, the fluorescence emission spectrum of DCG was measured by a fluorescence spectrophotometer.

Fe^3+^ detection: An aqueous solution of 1–100 mg/L FeCl_3_ was configured, and 0.5 mL was taken and added dropwise to the surface of DCG. Then, the fluorescence emission spectrum of DCG was measured by a fluorescence spectrophotometer.

### 4.6. Adsorption Performance Experiment

Adsorption kinetics: 0.1 g of DCG was immersed in 50 mL of FeCl_3_ aqueous solution (100 mg/L) and placed in a water bath thermostatic oscillator at 25 °C. The amount of adsorption was analyzed by a UV–visible spectrophotometer using sulfosalicylic acid as the chromogenic agent [56]. The current Fe^3+^ adsorption capacity (q_t_) of DCG was calculated using Equation (5):

(5) $$q_{t}=\frac{{(C}_{0}-{\;C}_{t})V}{m},$$

Adsorption thermodynamics: 0.1 g of DCG was immersed in 50 mL of FeCl_3_ aqueous solution (100 mg/L) and placed in a constant-temperature water bath shaker at 25 °C. The adsorption amount was analyzed by the same method as above after 72 h. The approximate equilibrium adsorption capacity (q_e_) was calculated using Equation (6):

(6) $$q_{e}=\frac{{(C}_{0}-{\;C}_{e})V}{m},$$

## Figures and Tables

**Table 1:** Young’s modulus of DCG.

Samples	1DCG	2DCG	3DCG	4DCG	5DCG
Young’s modulus (kPa)	35.3233	36.7557	93.6412	156	294

**Table 2:** Kinetic parameters of Fe^3+^ adsorption by DCG.

	Pseudo-First-Order Model			Pseudo-Second-Order Model		
Parameters Values	q_e_, cal (mg/g)	k_1_ (h^−1^)	R^2^	q_e_, cal (mg/g)	k_2_ (g·mg^−1^·h^−1^)	R^2^
	25.4363	0.48252	0.979	30.2633	0.01790	0.999

**Table 3:** Isotherm parameters of Fe^3+^ adsorption by DCG.

	Langmuir Isotherm			Freundlich Isotherm		
Parameters Values	q_m_, cal (mg/g)	k_L_ (L/mg)	R^2^	k_F_ (L/mg)	n	R^2^
	125.3011	0.00356	0.991	4.7004	2.2353	0.936

**Table 4:** Comparison of Fe^3+^ adsorption properties and detection limits between DCG and other materials.

Materials	q_max_ (mg/g)	LOD (mg/L)	Ref.
CMC-St/Al_2_O_3_	29.26	/	[51]
CMC-g-AMPS	33.65	/	[52]
FNH	98.3	62.5	[53]
CNC-*g*-PCysMA	60.0	/	[54]
TO-CNF	70.0	/	[54]
CP3	0.475	0.0269	[55]
DCG	125.30	0.6595	This study

## Data Availability

Data are contained within the article.

## Supplementary Materials

The following supporting information can be downloaded at https://www.mdpi.com/article/10.3390/gels10050285/s1, Figure S1: XPS spectrum of CMCS. Figure S2: Histograms of particle size distribution, TEM images, and HRTEM images of 1Si-CDs and 3Si-CDs. Figure S3: XPS spectra of CMC [57]. Figure S4: XRD spectra of CMC and DCG. Figure S5: Fluorescence emission lifetime decay diagram and fitting curve of Si-CDs [58]. Table S1–S4: Peak fitting data of XPS for 5Si-CDs, CMCS, 5DCG, and CMC.
