# Supplementary material for: Silicon-Doped Carbon Dots Crosslinked Carboxymethyl Cellulose Gel: Detection and Adsorption of Fe3+
Source: Gels. 2024 Apr 23;10(5):285. doi: 10.3390/gels10050285 (PMC11120848; doi:10.3390/gels10050285)
Supplement: Supplementary file 1 [file gels-10-00285-s001.zip › gels-2975399-supplementary.pdf]

Supporting Information

Silicon-Doped Carbon Dots Crosslinked Carboxymethyl Cellulose Gel: Detection and Adsorption of Fe^{3+}

Section 1：Si-CDs structural analysis

The full XPS spectrum of CMCS (Figure S1a) shows the presence of four elements, C, N, O, and Na. In the C 1s spectrum of CMCS (Figure S1b), C-C, C-N, C-O, C=O correspond to 284.80, 285.45, 286.55, 288.55 eV. In the N 1s spectrum of CMCS (Figure S1c), N-C, N-H correspond to 399.15, 399.95 eV. In the O 1s spectrum of CMCS (Figure S2d), O-H, O-C, O=C correspond to 531.35, 532.25, 533.25 eV[22].

Table S1. Peak fitting data of XPS for 5Si-CDs

Peak	Binding Energy (eV)	FWHM	Peak	Binding Energy (eV)	FWHM
C-O/C=O	287.43	1.30	O=C	533.99	1.40
C-N	285.68	0.99	O-Si	531.73	2.18
C-C	284.80	1.10	O-C	530.04	1.64
C-Si	283.74	1.45	Si-O	101.83	0.96
N-H	400.30	1.78	Si-N	101.01	0.95
N-C	399.23	1.12	Si-C	100.09	0.90
N-Si	398.08	1.33			

Table S2. Peak fitting data of XPS for CMCS

Peak	Binding Energy (eV)	FWHM	Peak	Binding Energy (eV)	FWHM
C=O	288.55	1.60	N-C	399.15	1
C-O	286.55	1.56	O=C	533.25	1.87
C-N	285.45	0.88	O-C	532.25	1.36
C-C	284.80	0.81	O-H	531.35	1.19
N-H	399.95	1.08			

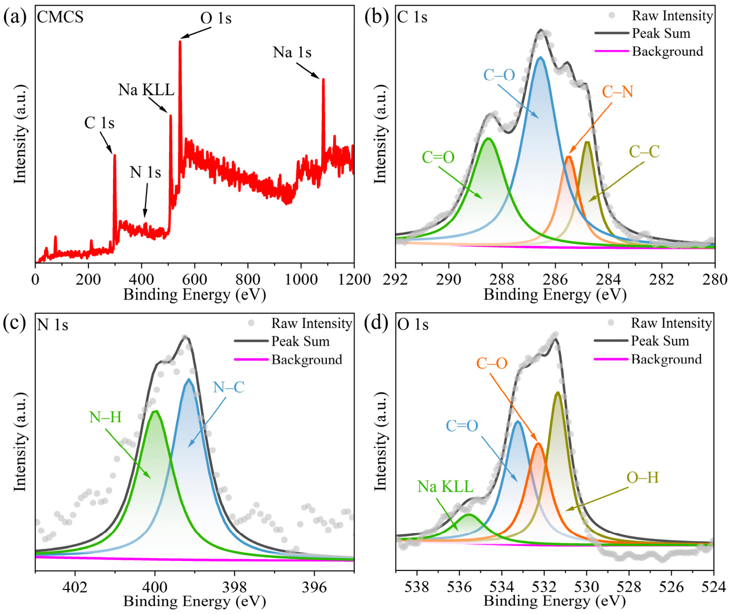

Figure S1. XPS spectra of CMCS. (a) Full spectrum, (b) C 1s fitting spectra, (c) N 1s fitting spectra, (d) O 1s fitting spectra.

(a)

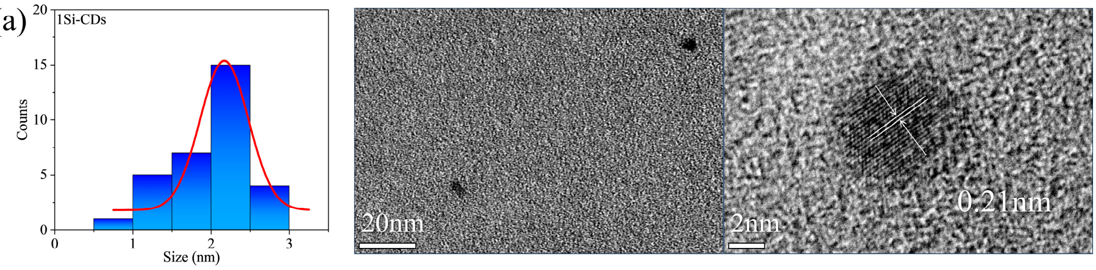

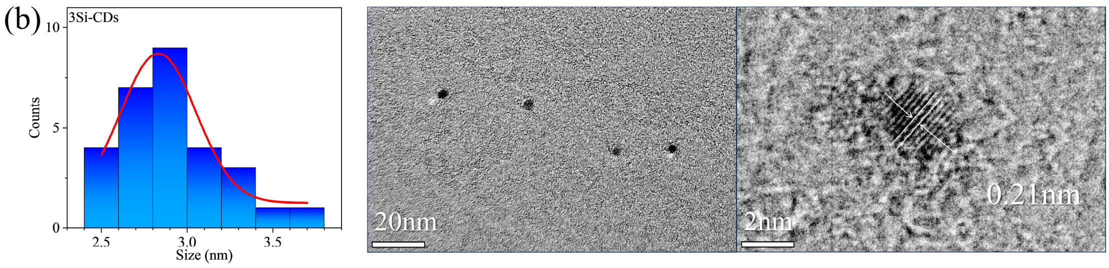

Figure S2. Histograms of particle size distribution, TEM images and HRTEM images of (a) 1Si-CDs, (b) 3Si-CDs.

Section 2： DCG structural analysis

The full XPS spectrum of CMC (Figure S2a) shows the presence of four elements, C, O, and Na. In the C 1s spectra of CMC (Figure S1b), C-C, C-O, C=O correspond to 284.80, 286.21, 287.73 eV. In the O 1s spectra of CMC (Figure S2c), O-H, O-C, O=C correspond to 530.98, 532.08, 533.23 eV[57].

Table S3. Peak fitting data of XPS for 5DCG

Peak	Binding Energy (eV)	FWHM	Peak	Binding Energy (eV)	FWHM
C-O/C=O	287.05	2.13	O=C	533.27	1.99
C-N	285.50	1.51	O-Si/O-C	532.00	1.54
C-C/C-Si	284.00	1.73	O-H	530.88	2.17
N-H	400.94	1.79	Si-O	102.30	1.62
NH-C=O	399.73	1.80	Si-N	101.76	0.94
N-Si	398.80	1.32	Si-C	100.97	0.94

Table S4. Peak fitting data of XPS for CMC

Peak	Binding Energy (eV)	FWHM	Peak	Binding Energy (eV)	FWHM
C=O	287.73	1.89	O=C	533.23	1.89
C-O	286.21	1.81	O-C	532.08	1.66
C-C	284.80	1.95	O-H	530.98	2.05

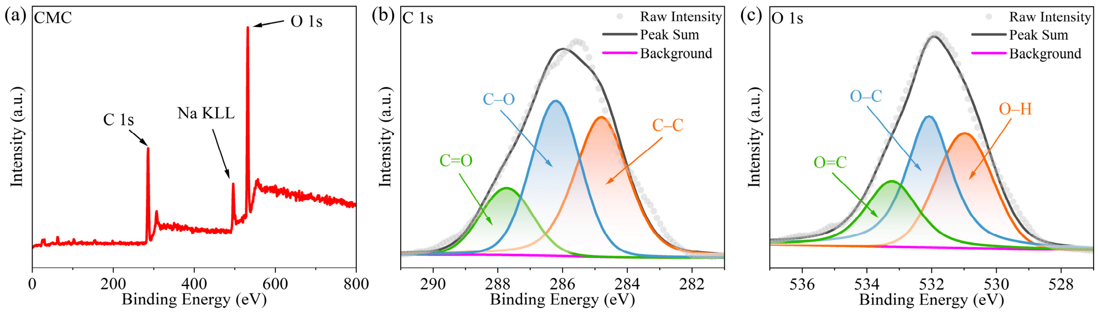

Figure S3. XPS spectra of CMC. (a) Full spectrum, (b) C 1s fitting spectra, (c) N 1s fitting spectra, (d) O 1s fitting spectra.

XRD tests: X-ray diffraction patterns were carried out using a CuKa (λ = 1.542 Å) radiation monochromator with a scanning range of 5-90°.

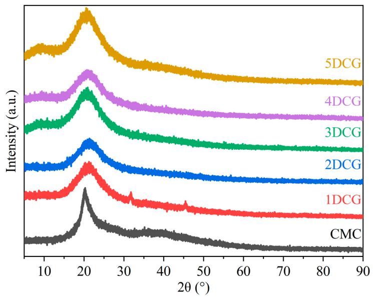

Figure S4. XRD spectra of CMC and DCG

Section 3 Analysis of Fluorescence Properties

Photoluminescence Spectrometer (FLS1000) was used to measure the fluorescence lifetime. The fluorescence decay curves of Si-CDs consistently adhere to a second-order exponential function, exhibiting correlation coefficients (R^2) greater than 0.97. The calculated fluorescence lifetimes consistently fall within the millisecond range[58].

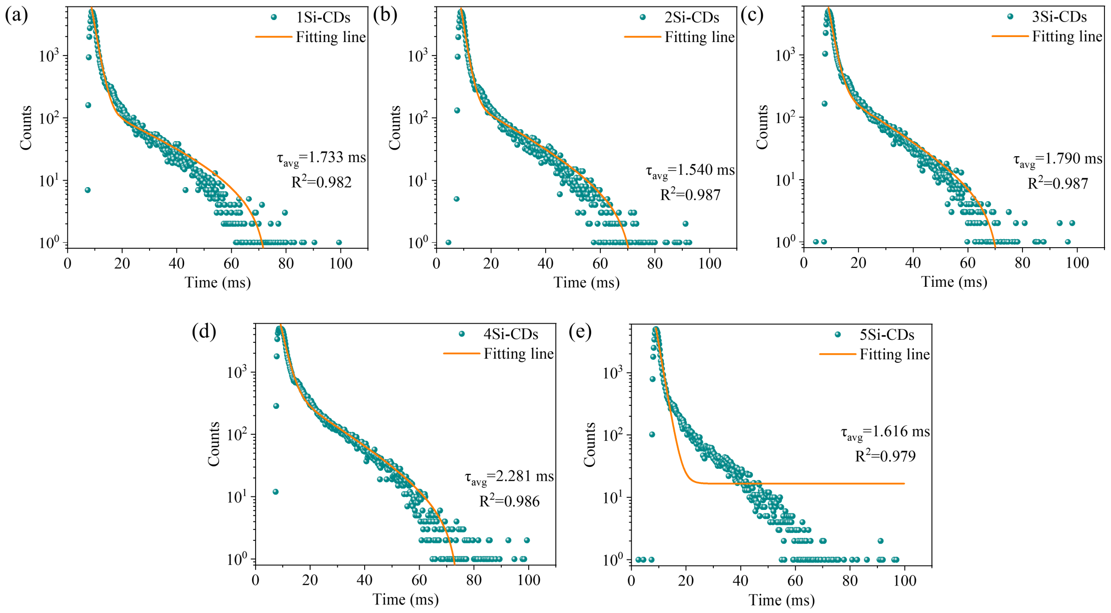

Figure S5. Fluorescence emission lifetime decay diagram and fitting curve of (a) 1Si-CDs, (b) 2Si-CDs, (c) 3Si-CDs, (d) 4Si-CDs and (e) 5Si-CDs

References

22. Li, Y., Z. Zhang, Z. Fu, D. Wang, C. Wang and J. Li. "Fluorescence response mechanism of green synthetic carboxymethyl chitosan-Eu^{3+} aerogel to acidic gases." *International Journal of Biological Macromolecules* 192 (2021): 1185-95. 10.1016/j.ijbiomac.2021.10.073. https://doi.org/10.1016/j.ijbiomac.2021.10.073.

57. Ye, J., B. Wang, J. Xiong and R. Sun. "Enhanced fluorescence and structural characteristics of carboxymethyl cellulose/Eu(III) nano-complex: Influence of reaction time." *Carbohydr Polym* 135 (2016): 57-63. 10.1016/j.carbpol.2015.08.063. http://dx.doi.org/10.1016/j.carbpol.2015.08.063.

58. Röding, M., S. J. Bradley, M. Nydén and T. Nann. "Fluorescence lifetime analysis of graphene quantum dots." *Journal of Physical Chemistry C* 118 (2014): 30282-90. 10.1021/jp510436r. https://doi.org/10.1021/jp510436r.
